# Key Odorant Identification Confirms 3-Oxododecanal as the Most Important Contributor to the Characteristic Aroma of Fresh Rhizomes and Leaves of *Houttuynia cordata*

**DOI:** 10.3390/foods14183147

**Published:** 2025-09-09

**Authors:** Zhenli Xu, Claudia Oellig, Walter Vetter, Martin Steinhaus, Stephanie Frank

**Affiliations:** 1 Leibniz Institute for Food Systems Biology at the Technical University of Munich, Lise-Meitner-Straße 34, 85354 Freising, Germany; z.xu.leibniz-lsb@tum.de; 2 Institute of Food Chemistry, University of Hohenheim, Garbenstraße 28, 70599 Stuttgart, Germany; claudia.oellig@uni-hohenheim.de (C.O.); walter.vetter@uni-hohenheim.de (W.V.)

**Keywords:** *Houttuynia cordata*, rhizome, leaf, 3-oxododecanal, myrcene, enantioselective odorant analysis, stable isotopically substituted odorant, odor activity value (OAV), aroma reconstitution, omission test

## Abstract

*Houttuynia cordata* is an Asian culinary herb with a characteristic fishy aroma. The most odor-active compounds, which had previously been identified by a comparative aroma extract dilution analysis applied to fresh rhizomes and leaves, were quantitated by GC–MS or GC–FID. Results revealed 23 and 22 compounds with odor activity values (OAVs) > 1, i.e., their concentrations exceeded their odor threshold concentrations, in rhizomes and leaves, respectively. Of these, myrcene (geranium leaf-like) and 3-oxododecanal (metallic, soapy, fishy) showed the highest OAVs. Aroma reconstitution and omission tests revealed that 3-oxododecanal is key to the characteristic fishy note. Results on the effect of tissue disruption suggested that 3-oxododecanal was already present in the intact *H. cordata* plant and released upon mechanical impact.

## 1. Introduction

*Houttuynia cordata* is a perennial, herbaceous, and stoloniferous plant in the Saururaceae family [1]. The plant consists of above-ground parts, including stems and leaves, and underground parts, which include roots and rhizomes. It is widely recognized as both a medicinal plant and a culinary herb. Fresh rhizomes and leaves are the main edible parts and are used in salads or as a topping in Asian countries such as China, Korea, and India [2]. *H*. *cordata* provides a range of vitamins, amino acids, and trace elements, such as zinc and copper [3]. The popularity of *H*. *cordata* rhizomes and leaves is associated with their distinct aroma, commonly characterized as fishy; however, there are slight differences in the overall aroma profiles of rhizomes and leaves.

To obtain a first insight into the compounds contributing to the characteristic aroma, we recently isolated the volatiles of fresh *H*. *cordata* rhizomes and leaves by automated solvent-assisted flavor evaporation (aSAFE) and screened the volatile fractions for odorants by comparative aroma extract dilution analysis (cAEDA) [4]. Results revealed 44 odor-active compounds in the rhizomes and 41 in the leaves. Flavor dilution (FD) factors ranged from 1 to 16,384. Thirty-eight of these odorants were perceived in both samples. A total of 44 odorants were successfully identified. High FD factors in the rhizomes and the leaves were determined for metallic, soapy, fishy smelling 3-oxododecanal (FD factors 16,384 and 4096), geranium leaf-like smelling myrcene (FD factors 2048 and 2048), metallic smelling (5*Z*)-octa-1,5-dien-3-one (FD factors 512 and 1024), resinous smelling *α*-pinene (FD factors 256 and 128), and coriander leaf-like smelling (2*E*)-dodec-2-enal (FD factors 128 and 128). In this study, the olfactory potential of 3-oxododecanal was recognized for the first time. Its metallic, soapy, and fishy odor in combination with its high FD factors suggested that 3-oxododecanal is the character impact odorant of *H*. *cordata*.

To support this hypothesis, the present study included (i) the quantitation of odor-active compounds detected with FD factors ≥ 32 in the cAEDA and the calculation of odor activity values (OAVs), (ii) sensory evaluation of aroma reconstitution models to verify the analytical data, and (iii) omission tests to assess the contribution of the individual odorants to the overall aroma.

## 2. Materials and Methods

### 2.1. Plant Materials

Fresh rhizomes and leaves were picked from potted *H*. *cordata* plants. More details on the plant materials can be found in our previous publication [4].

### 2.2. Reference Odorants

Reference odorants were purchased or synthesized as detailed recently [4].

### 2.3. Stable Isotopically Substituted Odorants

Compound (^2^H_2_)-**17** was purchased from Eptes (Vevey, Switzerland) and (^2^H_6–7_)-**40** from Merck (Darmstadt, Germany). Compounds (^2^H_3_)-**3** [5], (^2^H_2_)-**6** [6], (^13^C_8_)-**10** [7], (^2^H_4_)-**11** [8], (^2^H_2_)-**12** [9], (^2^H_3_)-**16** [8], (^2^H_3_)-**22** [10], (^2^H_2_)-**23** [9], (^13^C_2_)-**27** [11], (^2^H_3_)-**28** [12], (^2^H_2_)-**31** [13], (^2^H_3–6_)-**32** [14], (^2^H_2_)-**33** [15], (^2^H_2_)-**35** [15], (^2^H_2_)-**37** [16], (^13^C_6_)-**38** [17], (^2^H_3_)-**45** [18], (^2^H_3_)-**47** [19], and (^2^H_2_)-2-methoxy-4-propylphenol [20] were synthesized as detailed in the literature.

### 2.4. Miscellaneous Chemicals

Tridecane was purchased from Merck. Medium-chain triglycerides, type Miglyol 812, were obtained from Caelo (Hilden, Germany). Dichloromethane (CLN; Langenbach, Germany) was freshly distilled through a column (120 cm × 5 cm) packed with Raschig rings before use.

### 2.5. Gas Chromatography

A GC–FID instrument was used for the quantitation of *α*-pinene, myrcene, limonene, and 3-oxododecanal, and for enantioselective odorant analyses. GC–O analyses were performed with a GC–O/FID instrument. For GC–MS analyses, four different instruments were available: a GC–MS instrument with an ion trap mass spectrometer, a two-dimensional heart-cut GC–GC–MS instrument with an ion trap mass spectrometer, a two-dimensional heart-cut GC–GC–HRMS instrument with an orbitrap mass spectrometer, and a comprehensive two-dimensional GC×GC–MS instrument with a time-of-flight (TOF) mass spectrometer. Details on the individual GC instruments are available in the Supplementary Materials.

### 2.6. Enantioselective Odorant Analysis

For the enantioselective analysis of *α*-pinene (**2**), ethyl 2-methylbutanoate (**3**), and limonene (**8**), the GC–O/FID instrument, the GC–FID instrument, and the GC–GC–MS instrument were equipped with a chiral column BGB-176 (BGB Analytik; Lörrach, Germany). The elution order of the enantiomers was determined by a comparative analysis of the enantiomeric mixture and at least one of the individual enantiomers.

### 2.7. GC–MS Quantitations

Fresh rhizomes and leaves of *H*. *cordata* were cleaned with water and then cut into 0.5–1 cm strips with a ceramic knife within 5 min. Either cut rhizomes or leaves (1–40 g) were added to a mixture of saturated aqueous calcium chloride solution (10–60 mL), dichloromethane (100–200 mL), and stable isotopically substituted odorants serving as internal standards (cf. Supplementary Materials, Table S1). Depending on the expected target compound concentrations, amounts of the added internal standards varied between 0.01 and 20 µg. The mixture was homogenized under an argon atmosphere with a digital high-performance dispersing instrument, Ultra-Turrax T25 (IKA; Staufen, Germany). After further stirring under argon for 3 h, the organic phase was separated and dried over anhydrous sodium sulfate. Nonvolatiles were removed by aSAFE [21] at 40 °C using an open/closed time combination for the pneumatic valve of 0.2 s/10 s. The distillates were concentrated to 0.1–1.0 mL using a Vigreux column (60 cm × 1 cm) and a Bemelmans microdistillation device [22]. The concentrates were analyzed either with the GC–MS instrument (**10**, **11**, **17**, **22**, **23**, **31**, **33**, **40**, **42**, **45**), the two-dimensional heart-cut GC–GC–MS instrument (**3**, **6**, **35**), the two-dimensional heart-cut GC–GC–HRMS instrument (**12**, **16**, **27**, **28**, **32**, **37**, **38**), or the comprehensive two-dimensional GC×GC–MS instrument (**47**).

Peak areas of the analytes and the respective internal standards were collected from the extracted ion chromatograms using the quantifier ions detailed in the Supplementary Materials, Table S1. The concentration of each odorant in the rhizomes or leaves was calculated from the area counts of the analyte peak, the area counts of the internal standard peak, the amount of plant material used in the workup, and the amount of internal standard added, by employing a calibration line equation. To obtain the calibration line equation, solutions of the analyte and the respective internal standard were mixed in different concentration ratios and analyzed under the same conditions, followed by linear regression. The calibration line equations are available in the Supplementary Materials, Table S1. Individual concentrations and standard deviations can be found in the Supplementary Materials, Tables S2 and S3.

### 2.8. GC–FID Quantitations

For the quantitation of compounds **2**, **7**, **8**, and **36**, a water-cooled (12 °C) glass column (1 cm i.d.) was fitted with a plug of defatted cotton wool and sea sand (5 mm). Fresh rhizomes and leaves of *H. cordata* were cleaned with water and then cut into 0.5–1 cm strips with a ceramic knife within 5 min. Either cut rhizomes or leaves (5 g) were added to sodium sulfate (10 g) and dichloromethane (50 mL). The mixture was homogenized under an argon atmosphere with a digital high-performance dispersing instrument, Ultra-Turrax T25 (IKA). The homogenate was quantitatively transferred to the column using dichloromethane. After the sedimentation of the solids, a second layer of sea sand (1 cm) was applied. Elution was performed with dichloromethane (600 mL) at ~3 mL/min, and the eluate was collected in three 200 mL portions. Tridecane (708 μg) in dichloromethane (0.5 mL) was added to each portion as an internal standard. Each eluate portion was individually concentrated to 10 mL using a Vigreux column (60 cm × 1 cm). The concentrates were analyzed with the GC–FID instrument.

The concentrations of **2**, **7**, **8**, and **36** in the rhizomes and leaves were calculated from the area counts of the analyte peaks, the area counts of the internal standard peak, the amount of plant material used in the workup, the amount of internal standard added, and the response factors determined from the analysis of a reference compound mixture containing approximately equal concentrations of odorants **2**, **7**, **8**, and **36**, and the internal standard tridecane.

To evaluate the effect of tissue disruption on the concentration of **36**, whole rhizomes without cutting into strips were additionally analyzed using the same procedure.

### 2.9. Determination of Odor Threshold Concentrations (OTCs)

Orthonasal OTCs of (4*S*)-limonene (**8a**), geranyl acetate (**31**), (2*E*)-dodec-2-enal (**35**), and 3-oxododecanal (**36**) were determined in water according to the American Society for Testing and Materials (ASTM) standard practice for determination of odor and taste thresholds by a forced-choice ascending concentration series method of limits [23]. Assessors (5–7 males and 10–12 females, aged 24–60 years) were recruited from the trained panel of the Leibniz Institute for Food Systems Biology at the Technical University of Munich. The tests were conducted in separate booths in a room dedicated exclusively to sensory evaluations. The room temperature was 22 ± 2 °C. Further details, including the GC–O approach for the purity testing of the odorants before their use in the OTC determinations, are available in the literature [24].

### 2.10. Preparation of Aroma Reconstitution Models

The matrix for the aroma reconstitution models was a mixture of water (10 g), oxalic acid (0.008 g) [25], medium-chain triglycerides (0.05 g for rhizomes model, 0.04 g for leaves model) [26], and aqueous potassium hydroxide (2 mol/L; 0.11 g for rhizomes model, 0.09 g for leaves model). The alkaline solution was necessary to adjust the pH of the final aroma reconstitution models to 5.7 (rhizomes model) and 4.1 (leaves model), the values previously determined in the fresh plant materials. An individual ethanolic stock solution was prepared for each odorant for which an OAV ≥ 1 had been determined. The absence of odor-active impurities in the reference odorants was checked by GC–O [24]. Aliquots of the stock solutions were combined and diluted with the appropriate matrix solution to obtain final odorant concentrations in the models representing the concentrations previously determined in the fresh plant materials. Final ethanol concentrations were below 0.8 mL/kg. The complete aroma reconstitution models contained 23 odorants for the rhizomes and 22 for the leaves.

### 2.11. Quantitative Olfactory Profile Analyses

Quantitative olfactory profiles of fresh *H. cordata* rhizomes, fresh *H. cordata* leaves, and the corresponding aroma reconstitution models were obtained as described in our previous publication [4]. The ratings of all assessors were averaged by calculating the arithmetic mean.

### 2.12. Omission Tests

Incomplete aroma reconstitution models were prepared in the same way as the complete aroma reconstitution models, except that selected odorants were omitted. Each incomplete aroma reconstitution model was then tested orthonasally against the complete aroma reconstitution model in a three-alternative forced choice (3-AFC) test.

## 3. Results and Discussion

### 3.1. Odorant Concentrations and OAVs

Twenty-five odorants, for which the recent application of a cAEDA [4] had revealed an FD factor ≥ 32 in at least one of the two *H*. *cordata* samples, were quantitated in fresh rhizomes and leaves. Twenty-one of these odorants were quantitated by GC–MS, heart-cut GC–GC–MS, heart-cut GC–GC–HRMS, or comprehensive two-dimensional GC×GC–MS. Stable isotopically substituted odorants (Supplementary Materials, Table S1) served as internal standards to compensate for potential losses during the workup procedure. Due to high concentrations, this approach was not applicable for *α*-pinene (**2**), myrcene (**7**), limonene (**8**), and 3-oxododecanal (**36**); representative samples would have required uneconomically huge amounts of the standards. Therefore, the quantitation of these four odorants was performed by GC–FID after exhaustive extraction using tridecane as the internal standard. To ensure that the extraction of *α*-pinene, myrcene, limonene, and 3-oxododecanal was exhaustive, the column eluate was collected in successive portions. Each portion was spiked with the internal standard and then analyzed by GC–FID. As shown in Table 1, the first extract portion has already recovered all target compounds completely, thus proving exhaustive extraction.

**Figure 1:**
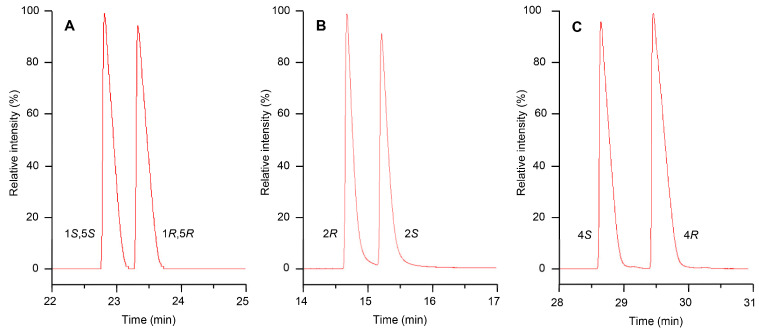
Enantiomeric separation of *α*-pinene (**A**), ethyl 2-methylbutanoate (**B**), and limonene (**C**) by GC–FID with a chiral column.

Enantiospecific concentrations of important chiral odorants were calculated from the sum of enantiomers as determined in the quantitation assays and the enantiomeric distribution as determined by GC with a chiral column (30% 2,3-dimethyl-6-*tert*-butyldimethylsilyl-*β*-cyclodextrin dissolved in 15% phenyl-, 85% methylpolysiloxane). The enantiomeric distributions of *α*-pinene (**2**), ethyl 2-methylbutanoate (**3**), and limonene (**8**) are listed in Table 2, and the chromatograms demonstrating the separation of the enantiomers are shown in Figure 1. In the case of *α*-pinene, the (1*S*,5*S*)-isomer predominated with 94% in the rhizomes, while in the leaves, with 46% (1*S*,5*S*)-isomer and 54% (1*R*,5*R*)-isomer, the ratio was almost racemic. Different enantiomeric ratios of *α*-pinene in different parts of the same plant have also been reported for other plants, e.g., *Pinus sylvestris* [27]. In both ethyl 2-methylbutanoate and limonene, the (*S*)-isomer clearly predominated. Ethyl 2-methylbutanoate was even enantiopure in the fresh rhizomes and leaves. Enantiopure ethyl (2*S*)-2-methylbutanoate has also been reported in some fruits, e.g., durian [28], and in fermented foods and beverages [29,30,31]. In limonene, the dominance of the (*S*)-isomer was 74% in the rhizomes and 98% in the leaves. Similar results were reported for white pepper with a 60% predominance of the (*S*)-limonene [32]. As pepper and *H*. *cordata* both belong to the *Piperales* order, this may reflect a similar biosynthesis. To the best of our knowledge, the enantiomeric distribution of *α*-pinene, ethyl 2-methylbutanoate, and limonene in *H*. *cordata* has not been reported so far.

The quantitation results are summarized in Table 3. The odorant concentrations ranged between ng/kg and g/kg. The highest concentrations in both fresh *H*. *cordata* rhizomes and leaves were determined for 3-oxododecanal (**36**) with 2.48 g/kg in the rhizomes and 1.34 g/kg in the leaves. High concentrations in the fresh rhizomes were additionally determined for (1*S*,5*S*)-*α*-pinene (**2a**; 143,000 μg/kg), myrcene (**7**; 111,000 μg/kg), (4*S*)-limonene (**8a**; 31,000 μg/kg), (4*R*)-limonene (**8b**; 10,900 μg/kg), (1*R*,5*R*)-*α*-pinene (**2b**; 9140 μg/kg), decanal (**17**; 5070 μg/kg), geranyl acetate (**31**; 2050 μg/kg), undecan-2-one (**22**; 2040 μg/kg), and butanoic acid (**23**; 1660 μg/kg). In the fresh leaves, myrcene (**7**; 140,000 μg/kg), decanal (**17**; 13,600 μg/kg), (1*R*,5*R*)-*α*-pinene (**2b**; 7150 μg/kg), (1*S*,5*S*)-*α*-pinene (**2a**; 6090 μg/kg), and geranyl acetate (**31**; 4960 μg/kg) also showed high concentrations. In the rhizomes, a total of ten compounds showed concentrations above 1 mg/kg; in the leaves, only six compounds reached this concentration level.

To obtain information about the odor activity of the individual odorants, OAVs were calculated as the ratio of the odorant concentration in the plant material and the orthonasal OTC in water. OTCs in water were used because *H*. *cordata* tissue contains >90% water [25]. Calculation results (Table 3) revealed 23 and 22 odorants with OAVs ≥ 1 in the rhizomes and the leaves, respectively. The highest OAVs were calculated for myrcene (**7**; OAVs 93,000 and 120,000) and 3-oxododecanal (**36**; OAVs 27,000 and 15,000) in both plant parts. Numerous studies have mentioned high concentrations of myrcene and 3-oxododecanal in *H*. *cordata* [35,36,37,38,39]; however, there has been no comparison of fresh rhizomes and leaves based on OAVs, and the current study is the first to report the OTC of 3-oxododecanal. Although the OAV of myrcene was higher than that of 3-oxododecanal, results suggested 3-oxododecanal, due to its specific odor, as the character impact compound of *H*. *cordata*.

Three additional odorants showed OAVs ≥ 1000 in the rhizomes, namely (4*S*)-limonene (**8a**; OAV 3900), (5*Z*)-octa-1,5-dien-3-one (**12**; OAV 3400), and (1*R*,5*R*)-*α*-pinene (**2b**; OAV 1000). In the leaves, two more odorants had OAVs ≥ 1000, namely (5*Z*)-octa-1,5-dien-3-one (**12**; OAV 3100) and decanal (**17**; OAV 1500). (5*Z*)-Octa-1,5-dien-3-one had not been quantitated in *H*. *cordata* before. In contrast, high amounts of limonene and *α*-pinene in *H*. *cordata* have previously been reported [35,38,40,41]. However, in no case were the enantiomeric distributions considered. For odor activity evaluation, it is essential to separately quantitate the enantiomers, as their OTCs are usually not the same. For example, the OTCs of (1*S*,5*S*)- and (1*R*,5*R*)-*α*-pinene differ by a factor of ~20.

The OAV range of <1000, but >100 included six odorants in the rhizomes, and seven in the leaves. The odorants in the rhizomes were (1*S*,5*S*)-*α*-pinene (**2a**; OAV 840), (4*R*)-limonene (**8b**; OAV 840), (2*E*)-dodec-2-enal (**35**; OAV 580), decanal (**17**; OAV 550), oct-1-en-3-one (**11**; OAV 290), and (3*Z*)-hex-3-enal (**6**; OAV 130). The odorants in the leaves were (3*Z*)-hex-3-enal (**6**; OAV 850), (1*R*,5*R*)-*α*-pinene (**2b**; OAV 790), (2*E*)-dodec-2-enal (**35**; OAV 420), oct-1-en-3-one (**11**; OAV 340), ethyl (2*S*)-2-methylbutanoate (**3b**; OAV 200), geranyl acetate (**31**; OAV 160), and (*E*)-*β*-damascenone (**32**; OAV 140). The OAVs of (1*S*,5*S*)-*α*-pinene and (4*R*)-limonene were significantly higher in the rhizomes than in the leaves. For (4*R*)-limonene, the difference was more than 2500-fold. For (4*S*)-limonene, the factor was 160. In contrast, the OAV of ethyl (2*S*)-2-methylbutanoate was higher in the leaves than in the rhizomes. However, most of the odorants listed in Table 3 showed only little OAV differences between the two different parts of the plant.

### 3.2. Aroma Reconstitution

**Figure 2:**
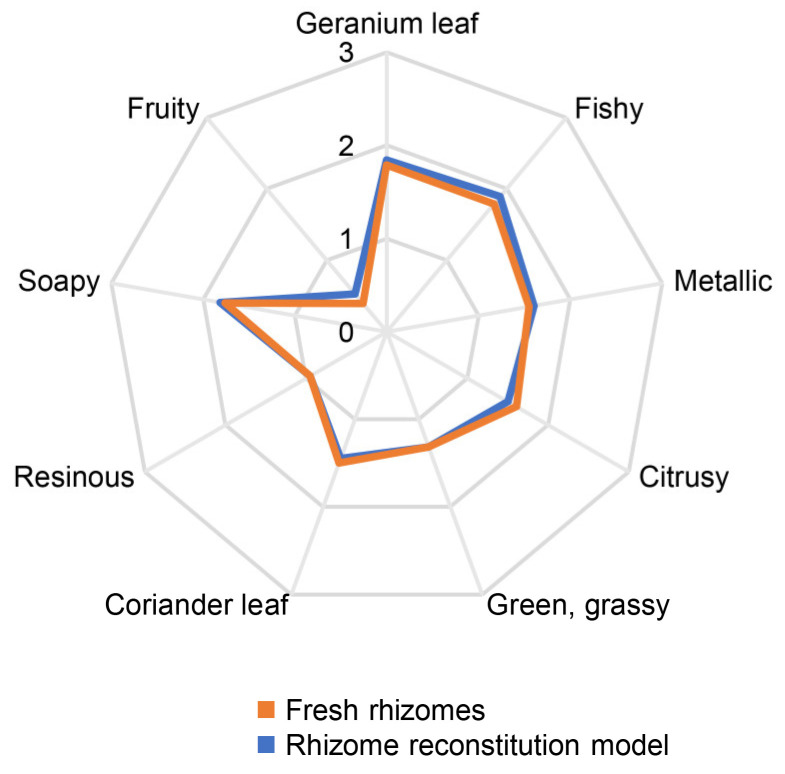
Quantitative olfactory profile of fresh rhizomes of *H*. *cordata* in comparison to the quantitative olfactory profile of the aroma reconstitution model (23 odorants, OAVs ≥ 1). Assessors rated the intensity of each descriptor on a scale ranging from 0 to 3 with 0.5 increments and 0 = not detectable, 1 = weak, 2 = moderate, and 3 = strong.

**Figure 3:**
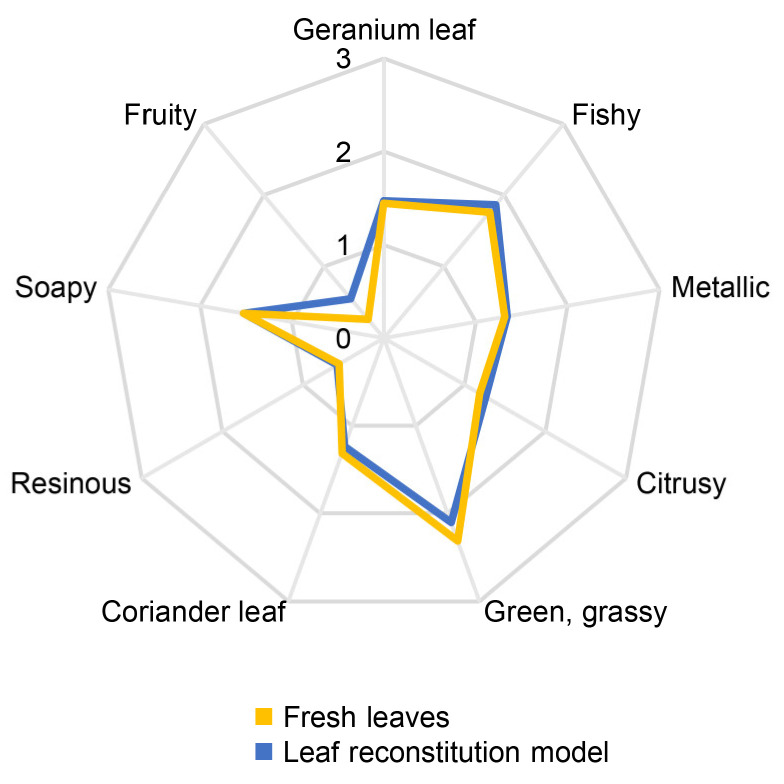
Quantitative olfactory profile of fresh leaves of *H*. *cordata* in comparison to the quantitative olfactory profile of the aroma reconstitution model (22 odorants, OAVs ≥ 1). Assessors rated the intensity of each descriptor on a scale ranging from 0 to 3 with 0.5 increments and 0 = not detectable, 1 = weak, 2 = moderate, and 3 = strong.

To verify the completeness and accuracy of the collected data, aroma reconstitution experiments were conducted. An aroma reconstitution model was prepared for each of the two plant materials containing all odorants with OAVs ≥ 1 in the previously determined concentrations (cf. Table 3), i.e., 23 for the rhizomes and 22 for the leaves. A trained sensory panel evaluated the aroma reconstitution models in direct comparison with the freshly harvested plant materials. The resulting quantitative olfactory profiles (Figure 2 and Figure 3) revealed excellent agreements between the models and the plant materials, thus providing evidence that all major aroma-contributing compounds in fresh *H. cordata* rhizomes and leaves were correctly identified and quantitated. In particular, the models reflected the characteristic aroma differences between the rhizomes and the leaves, e.g., the higher intensity of the green, grassy note in the leaves compared to the rhizomes.

### 3.3. Key Odorants

To assess the contribution of the individual odorants to the overall aroma, omission tests were performed. If an omission test results in a significant difference between the complete and the incomplete aroma reconstitution model, the omitted compound is considered a key odorant in the food under consideration [42].

In the first set of experiments, omitted compounds were selected according to their OAVs. For fresh rhizomes and leaves, the omission of compounds with OAVs ≤ 100 did not lead to a significant difference between the complete and incomplete aroma reconstitution models (Table 4 and Table 5, tests OR-A1 and OL-A1). Omitting all compounds with OAVs < 850 yielded the same result (tests OR-A2 and OL-A2). Thus, only five odorants were sufficient to imitate the overall aroma of fresh *H. cordata* rhizomes and leaves.

In the second set of experiments, one of these five odorants was omitted individually. For the rhizomes, the tests OR-B1 and OR-B3 to OR-B5 yielded no significant difference between the complete and incomplete aroma reconstitution models. In contrast, the test OR-B2, including the omission of 3-oxododecanal (**36**), resulted in a very highly significant difference. Consequently, 3-oxododecanal was demonstrated to be the key odorant in fresh rhizomes of *H. cordata*. For the leaves, the tests OL-B3 to OL-B5 yielded no significant difference between the complete and incomplete aroma reconstitution models; however, tests OL-B1, including the omission of myrcene (**7**), and OL-B2, including the omission of 3-oxododecanal (**36**), resulted in a very highly significant difference between the complete and incomplete aroma reconstitution models, identifying myrcene and 3-oxododecanal as the key odorants in fresh leaves of *H. cordata*.

### 3.4. Effect of Tissue Disruption on 3-Oxododecanal

To answer the question of whether 3-oxododecanal (**36**) was already present in the intact plant or formed by enzymatic reactions during injury, the influence of tissue disruption on the 3-oxododecanal concentration in fresh rhizomes was studied. The concentrations of *α*-pinene (**2**), myrcene (**7**), and limonene (**8**) were determined in parallel. Since it is known that these terpenes are not enzymatically formed upon injury, they served as internal anchors to visualize methodological and biological variance. Quantitation of the target compounds in the intact rhizomes was accomplished as described in the Materials and Methods section after homogenizing the carefully harvested fresh intact rhizomes with anhydrous sodium sulfate, an approach known to effectively inhibit enzymatic activity [43]. A second sample, representing the sample with some tissue disruption, was prepared by cutting the fresh rhizomes into small strips within 5 min, before homogenization with anhydrous sodium sulfate. The concentrations of all four compounds (Table 6) were slightly higher in the whole rhizomes than in the rhizome strips. However, the concentration differences between the two samples were within the standard deviation ranges. The concentration of 3-oxododecanal was 2,760,000 μg/kg ± 250,000 μg/kg in the whole rhizomes and 2,480,000 μg/kg ± 160,000 μg/kg in the rhizome strips. Thus, data suggested that 3-oxododecanal was not enzymatically formed after tissue disruption. An enzymatic reaction would have led to a significantly higher concentration in the rhizome strips compared to the whole rhizomes. For example, (3*Z*)-hex-3-enal, known to be formed upon tissue disruption from linolenic acid via the lipoxygenase/hydroperoxide lyase pathway, showed a concentration more than 50-fold higher in curry leaf strips than in the whole leaves [34]. Obviously, 3-oxododecanal was already present in the intact *H. cordata* tissue and released upon mechanical impact. The secretion of aldehydes and ketones from *H. cordata* glands, including 3-oxododecanal, and their roles in the defense against herbivores and microbial pathogens have already been reported [44].

## 4. Conclusions

This work bridged some gaps in the literature. It revealed, for the first time, the key odorants in fresh *H. cordata* rhizomes and leaves and confirmed 3-oxododecanal as causative for the characteristic fishy note. 3-Oxododecanal was already present in the intact *H. cordata* tissue and released, but not formed, upon mechanical impact.

## Figures and Tables

**Table 1:** Exhaustive Extraction of *α*-Pinene, Myrcene, Limonene, and 3-Oxododecanal from Fresh Rhizomes and Leaves of *H*. *cordata.*

Odorant	Replicate ^1^	Extract Portion ^2^	Concentration (μg/kg) ^3^			Concentration (%) ^4^	
			Rhizomes	Leaves		Rhizomes	Leaves
*α*-pinene	1	1	143,097	12,628		100	100
		2	0	0		0	0
		3	0	0		0	0
	2	1	152,590	11,728		100	100
		2	0	0		0	0
		3	0	0		0	0
	3	1	161,436	15,378		100	100
		2	0	0		0	0
		3	0	0		0	0
myrcene	1	1	103,576	139,970		100	100
		2	0	0		0	0
		3	0	0		0	0
	2	1	94,347	137,496		100	100
		2	0	0		0	0
		3	0	0		0	0
	3	1	135,502	141,241		100	100
		2	0	0		0	0
		3	0	0		0	0
limonene	1	1	40,709	230		100	100
		2	0	0		0	0
		3	0	0		0	0
	2	1	40,055	169		100	100
		2	0	0		0	0
		3	0	0		0	0
	3	1	45,106	178		100	100
		2	0	0		0	0
		3	0	0		0	0
3-oxododecanal	1	1	2,377,222	1,266,578		100	100
		2	0	0		0	0
		3	0	0		0	0
	2	1	2,406,408	1,304,849		100	100
		2	0	0		0	0
		3	0	0		0	0
	3	1	2,662,308	1,453,164		100	100
		2	0	0		0	0
		3	0	0		0	0

^1^ All analyses were carried out in triplicate (biological replicates). ^2^ The column eluate was collected in three portions of 200 mL each. ^3^ Concentration in μg/kg fresh plant material. ^4^ Percentage of the target odorant in each of the three eluate portions.

**Table 2:** Enantiomeric Distribution of Important Chiral Odorants in Fresh Rhizomes and Leaves of *H*. *cordata*.

Odorant	Odor ^1^	RI ^2^	Enantiomeric Distribution ^3^ (%) in	
		BGB-176	Rhizomes	Leaves
(1*S*,5*S*)-*α*-pinene	resinous	987	94	46
(1*R*,5*R*)-*α*-pinene	resinous	994	6	54
				
ethyl (2*R*)-2-methylbutanoate	fruity	873	0	0
ethyl (2*S*)-2-methylbutanoate	fruity	882	100	100
				
(4*S*)-limonene	geranium leaf, citrusy	1065	74	98
(4*R*)-limonene	citrusy	1076	26	2

^1^ Odor as perceived at the sniffing port during GC–O. ^2^ Retention index; calculated from the retention time of the odorant and the retention times of adjacent *n*-alkanes by linear interpolation. ^3^ Mean of biological triplicates.

**Table 3:** Concentrations and OAVs of Major Odorants in Fresh Rhizomes and Leaves of *H*. *cordata*.

No. ^1^	Odorant ^2^	Concentration ^3^ (µg/kg)		OTC ^4^ (μg/kg)	OAV ^5^	
		Rhizomes	Leaves		Rhizomes	Leaves
**7**	myrcene	111,000	140,000	1.2 ^6^	93,000	120,000
**36**	3-oxododecanal	2,480,000	1,340,000	92 ^7^	27,000	15,000
**8a**	(4*S*)-limonene ^8^	31,000	188	8.0 ^7^	3900	24
**12**	(5*Z*)-octa-1,5-dien-3-one	1.16	1.05	0.00034 ^6^	3400	3100
**17**	decanal	5070	13,600	9.3 ^6^	550	1500
**2b**	(1*R*,5*R*)-*α*-pinene ^8^	9140	7150	9.0 ^9^	1000	790
**6**	(3*Z*)-hex-3-enal	15.1	102	0.12 ^6^	130	850
**2a**	(1*S*,5*S*)-*α*-pinene ^8^	143,000	6090	170 ^6^	840	36
**8b**	(4*R*)-limonene ^8^	10,900	3.84	13 ^6^	840	<1
**35**	(2*E*)-dodec-2-enal	128	91.9	0.22 ^7^	580	420
**11**	oct-1-en-3-one	4.7	5.38	0.016 ^6^	290	340
**3b**	ethyl (2*S*)-2-methylbutanoate ^8^	0.149	1.64	0.0080 ^6^	19	200
**31**	geranyl acetate	2050	4960	31 ^7^	66	160
**32**	(*E*)-*β*-damascenone	0.109	0.826	0.0060 ^6^	18	140
**22**	undecan-2-one	2040	686	24 ^10^	85	29
**28**	3-methylnonane-2,4-dione	0.885	2.41	0.046 ^6^	19	52
**33**	geraniol	40.5	4.23	1.1 ^6^	37	3.8
**42**	eugenol	59.1	16.1	1.8 ^6^	33	9
**45**	*trans*-isoeugenol	15.9	13.2	0.716	22	19
**16**	3-(methylsulfanyl)propanal	7.18	0.165	0.43 ^6^	17	<1
**10**	octanal	39.9	24.1	3.4 ^6^	12	7.1
**27**	(2*E*,4*E*)-nona-2,4-dienal	<0.025	0.286	0.046 ^6^	<1	6.2
**40**	4-methylphenol	6.42	16.1	3.9 ^6^	1.6	4.1
**47**	vanillin	149	13.9	53 ^6^	2.8	<1
**38**	4-methoxybenzaldehyde	1.44	4.04	2.6 ^6^	<1	1.6
**23**	butanoic acid	1660	352	2400 ^6^	<1	<1
**37**	*trans*-4,5-epoxy-(2*E*)-dec-2-enal	0.069	0.141	0.22 ^6^	<1	<1

^1^ Numbering according to our previous paper [4]. ^2^ Odorants in order of decreasing OAVs. ^3^ Mean of biological duplicates or triplicates; individual values and standard deviations are available in the Supplementary Materials, Tables S2 and S3. ^4^ Orthonasal odor threshold concentration in water. ^5^ Odor activity value; calculated as a ratio of concentration to odor threshold concentration. ^6^ Data taken from the Leibniz-LSB@TUM Odorant Database [33]. ^7^ Data obtained in the current study. ^8^ Concentrations of individual enantiomers were calculated from the concentration of the sum of enantiomers as obtained in the quantitation assays and the enantiomeric distribution depicted in Table 2. ^9^ Data from literature [34]. ^10^ Data from literature [17].

**Table 4:** Omission Tests Applied to the Aroma Reconstitution Model of the Fresh Rhizomes.

Test	Odorants in the Incomplete Model ^1^	Correct Answers/ Assessors ^2^	*p*-Value (%)	Level of Significance
OR-A1	**7**, **36**, **8a**, **12**, **2b**, **2a**, **8b**, **35**, **17**, **11**, **6**	6/16	45	not significant (*p* > 5%)
OR-A2	**7**, **36**, **8a**, **12**, **2b**	8/16	13	not significant (*p* > 5%)
OR-B1	**36**, **8a**, **12**, **2b**	7/19	46	not significant (*p* > 5%)
OR-B2	**7**, **8a**, **12**, **2b**	16/19	0.00073	very highly significant (*p* < 0.1%)
OR-B3	**7**, **36**, **12**, **2b**	7/19	46	not significant (*p* > 5%)
OR-B4	**7**, **36**, **8a**, **2b**	9/19	15	not significant (*p* > 5%)
OR-B5	**7**, **36**, **8a**, **12**	10/19	6.5	not significant (*p* > 5%)

^1^ The complete model consisted of all 23 odorants with OAVs ≥ 1, i.e., odorants **2a**, **2b**, **3b**, **6**, **7**, **8a**, **8b**, **10**, **11**, **12**, **16**, **17**, **22**, **28**, **31**, **32**, **33**, **35**, **36**, **40**, **42**, **45**, **47**. ^2^ Number of correct answers resulting from the 3-AFC test and total number of assessors participating.

**Table 5:** Omission Tests Applied to the Aroma Reconstitution Model of the Fresh Leaves.

Test	Odorants in the Incomplete Model ^1^	Correct Answers/ Assessors ^2^	*p*-Value (%)	Level of Significance
OL-A1	**7**, **36**, **12**, **17**, **6**, **2b**, **35**, **11**, **3b**, **31**, **32**	5/16	66	not significant (*p* > 5%)
OL-A2	**7**, **36**, **12**, **17**, **6**	8/15	8.8	not significant (*p* > 5%)
OL-B1	**36**, **12**, **17**, **6**	14/20	0.088	very highly significant (*p* < 0.1%)
OL-B2	**7**, **12**, **17**, **6**	18/20	0.000023	very highly significant (*p* < 0.1%)
OL-B3	**7**, **36**, **17**, **6**	6/20	70	not significant (*p* > 5%)
OL-B4	**7**, **36**, **12**, **6**	7/20	52	not significant (*p* > 5%)
OL-B5	**7**, **36**, **12**, **17**	9/20	19	not significant (*p* > 5%)

^1^ The complete model consisted of all 22 odorants with OAVs ≥ 1, i.e., odorants **2a**, **2b**, **3b**, **6**, **7**, **8a**, **10**, **11**, **12**, **17**, **22**, **27**, **28**, **31**, **32**, **33**, **35**, **36**, **38**, **40**, **42**, **45**. ^2^ Number of correct answers resulting from the 3-AFC test and total number of assessors participating.

**Table 6:** Concentrations of Selected Odorants in Fresh Rhizomes of *H*. *cordata*: Effect of Tissue Disruption.

Odorant	Concentration (μg/kg)	
	Rhizome Strips ^1^	Whole Rhizomes ^2^
*α*-pinene	152,000 ± 9200	162,000 ± 19,000
myrcene	111,000 ± 22,000	129,000 ± 33,000
limonene	42,000 ± 2700	45,000 ± 5200
3-oxododecanal	2,480,000 ± 160,000	2,760,000 ± 250,000

^1^ Data taken from Table 1. ^2^ Mean of biological triplicates ± standard deviation.

## Data Availability

The original contributions presented in this study are included in this article/Supplementary Materials. Further inquiries can be directed to the corresponding authors.

## Supplementary Materials

The following supporting information can be downloaded at https://www.mdpi.com/article/10.3390/foods14183147/s1, Additional information on GC instruments; Table S1: stable isotopically substituted internal standards, quantifier ions, and calibration lines used in the quantitation assays; Table S2: concentrations of important odorants in fresh rhizomes of *H. cordata*; Table S3: concentrations of important odorants in fresh leaves of *H. cordata*. References [45,46] are cited in the Supplementary Materials.

## Abbreviations and Nomenclature

The following abbreviations and trivial compound names are used in this manuscript:

3-AFC	Three-alternative forced choice
aSAFE	Automated solvent-assisted flavor evaporation
cAEDA	Comparative aroma extract dilution analysis
(*E*)-*β*-damascenone	(2*E*)-1-(2,6,6-trimethylcyclohexa-1,3-dien-1-yl)but-2-en-1-one
*trans*-4,5-epoxy-(2*E*)-dec-2-enal	(2*E*)-3-[(2*R*,3*R*)/(2*S*,3*S*)-3-pentyloxiran-2-yl]prop-2-enal
eugenol	2-methoxy-4-(prop-2-en-1-yl)phenol
FD	Flavor dilution
FID	Flame ionization detector
GC	Gas chromatography
GC–O	Gas chromatography–olfactometry
geraniol	(2*E*)-3,7-dimethylocta-2,6-dien-1-ol
geranyl acetate	(2*E*)-3,7-dimethylocta-2,6-dien-1-yl acetate
HR	High resolution
i.d.	Inner diameter
*trans*-isoeugenol	2-methoxy-4-[(1*E*)-prop-1-en-1-yl]phenol
limonene	1-methyl-4-(prop-1-en-2-yl)cyclohex-1-ene
MS	Mass spectrometry
myrcene	7-methyl-3-methylideneocta-1,6-diene
OAV	Odor activity value
OTC	Odor threshold concentration
*α*-pinene	2,6,6-trimethylbicyclo[3.1.1]hept-2-ene
RI	Retention index
TOF	Time-of-flight
vanillin	4-hydroxy-3-methoxybenzaldehyde
