# Supplementary material for: Key Odorant Identification Confirms 3-Oxododecanal as the Most Important Contributor to the Characteristic Aroma of Fresh Rhizomes and Leaves of Houttuynia cordata
Source: Foods. 2025 Sep 9;14(18):3147. doi: 10.3390/foods14183147 (PMC12468953; doi:10.3390/foods14183147)
Supplement: Supplementary file 1 [file foods-14-03147-s001.zip › foods-3843052-supplementary.pdf]

Supplementary Materials

Key Odorant Identification Confirms 3-Oxododecanal as the Most Important Contributor to the Characteristic Aroma of Fresh Rhizomes and Leaves of *Houttuynia cordata*

Zhenli Xu,[1,2] Claudia Oellig,[2] Walter Vetter,[2] Martin Steinhaus,[1,*] and Stephanie Frank[1,*]

[1] Leibniz Institute for Food Systems Biology at the Technical University of Munich,
Lise-Meitner-Straße 34, 85354 Freising, Germany
[2] Institute of Food Chemistry, University of Hohenheim,
Garbenstraße 28, 70599 Stuttgart, Germany

*E-mail: m.steinhaus.leibniz-lsb@tum.de, s.frank.leibniz-lsb@tum.de

Overview

Additional Information on GC Instruments

GC–FID Instrument

GC–O/FID Instrument

GC–MS Instrument

Heart-Cut GC–GC–MS Instrument

Heart-Cut GC–GC–HRMS Instrument

Comprehensive Two-Dimensional GC×GC–MS Instrument

Additional Tables

Table S1. Stable Isotopically Substituted Internal Standards, Quantifier Ions, and Calibration Lines Used in the Quantitation Assays

Table S2. Concentrations of Important Odorants in Fresh Rhizomes of *H. cordata*

Table S3. Concentrations of Important Odorants in Fresh Leaves of *H. cordata*

GC–FID Instrument

A Trace Gas Chromatograph Ultra (Thermo Fisher Scientific; Dreieich, Germany) was equipped with a cold on-column injector and a flame ionization detector (FID; 250 °C base temperature). The column was either a DB-FFAP column, 30 m × 0.32 mm i.d., 0.25 µm film thickness (Agilent; Waldbronn, Germany) or a BGB-176 column, 30 m × 0.25 mm i.d., 0.25 µm film thickness (BGB Analytik; Lörrach, Germany). The carrier gas was helium at 65 kPa (DB-FFAP) and 130 kPa (BGB-176) constant pressure. The injection volume was 2 µL. The initial oven temperature was 40 °C for 2 min. Then the temperature was ramped at 6 °C/min to 230 °C (DB-FFAP) or at 2 °C/min to 200 °C (BGB-176), which was held for 5 min. The FID was connected to a computer. Chromatogram recording and peak area count calculation were performed by ChromQuest 5.0 (Thermo Fisher Scientific).

GC–O/FID Instrument

A Trace Gas Chromatograph Ultra (Thermo Fisher Scientific) was equipped with a cold on-column injector, an FID, and a sniffing-port custom-made from aluminum as detailed in *J. Agric. Food Chem*. **2008**, *56*, 4120–4127. The column was a BGB-176 column, 30 m × 0.25 mm i.d., 0.25 µm film thickness (BGB Analytik). The carrier gas was helium at 130 kPa constant pressure. The injection volume was 2 µL. The oven temperature program was identical to the one for the BGB-176 column detailed in the GC–FID instrument section. A Y-shaped glass splitter connected the end of the column with two uncoated but deactivated fused silica capillaries, each 50 cm × 0.25 mm i.d., which delivered the column effluent in two equal parts to the FID (250 °C base temperature) and the sniffing port (230 °C base temperature), respectively. For GC–O analysis, a trained assessor placed the nose directly above the sniffing port and evaluated the effluent. Whenever an odor was detected, the position, as well as the odor quality, were marked in the FID chromatogram. For each odorant, a retention index was calculated by linear interpolation from its retention time and the retention times of adjacent *n*-alkanes as detailed in *J. Chromatogr. A*. **1963**, *11*, 463–471.

GC–MS Instrument

A 7890B gas chromatograph (Agilent) was equipped with a GC 80 autosampler and a multimode injector. The column was a DB-FFAP column, 30 m × 0.25 mm i.d., 0.25 µm film thickness (Agilent). The carrier gas was helium at 1.0 mL/min constant flow. The injection volume was 1 µL or 2 µL. The oven temperature program was identical to the one for the DB-FFAP column detailed in the GC–FID instrument section. The GC was connected to a Saturn 240 ion trap mass spectrometer (Agilent) operated in the chemical ionization (CI) mode with methanol as reagent gas and a scan range of *m/z* 60–250. Data were analyzed with the MS Workstation software (Agilent).

Heart-Cut GC–GC–MS Instrument

A Trace GC Ultra (Thermo Fisher Scientific) was equipped with a Combi PAL autosampler (CTC Analytics; Zwingen, Switzerland), a cold on-column injector, an FID (250 °C base temperature), and a custom-made sniffing port (cf. *J. Agric. Food Chem.* **2008**, *56*, 4120–4127; 230 °C base temperature). The column was a DB-FFAP column, 30 m × 0.32 mm i.d., 0.25 µm film thickness (Agilent). The carrier gas was helium at 110 kPa constant pressure. The injection volume was 2 µL. The initial oven temperature was 40 °C for 2 min. Then the temperature was ramped at 6 °C/min to 230 °C, which was held for 5 min. The end of the column was connected to a moving column stream switching (MCSS) device (Thermo Fisher Scientific) used for heart-cutting. The MCSS device directed the eluate of the column via uncoated but deactivated fused silica capillaries (0.32 mm i.d.) time-programmed either simultaneously to the FID and the sniffing port used as monitor detectors or to a second GC column, which was either a DB-1701 column, 30 m ×

0.25 mm i.d., 0.25 µm film thickness (Agilent) or a BGB-176 column, 30 m × 0.25 mm i.d., 0.25 µm film thickness (BGB Analytik). The second column was installed in a separate gas chromatograph (CP 3800; Varian; Darmstadt, Germany). The capillary to the second column first passed through a heated (250 °C) hose connecting the two gas chromatographs and then through a liquid nitrogen-cooled trap installed inside the oven of the second gas chromatograph. The trap was used to refocus the heart-cut. The initial temperature of the second oven was 40 °C for 2 min. Then the temperature was ramped at 6 °C/min to 240 °C (DB-1701) or at 2 °C/min to 200 °C (BGB-176), which was held for 5 min. The end of the second column was connected to a Saturn 2200 ion trap mass spectrometer (Varian) operated in the CI mode with methanol as reagent gas and a scan range *m/z* 60–250. Data were analyzed with the MS Workstation software (Agilent).

Heart-Cut GC–GC–HRMS Instrument

A Trace 1310 gas chromatograph (Thermo Fisher Scientific) was equipped with a TriPlus RSH autosampler, a programmed temperature vaporizing (PTV) injector, an FID (250 °C base temperature), and a custom-made sniffing port (cf. *J. Agric. Food Chem*. **2008**, *56*, 4120–4127; 230 °C base temperature). The column was a DB-FFAP column, 30 m × 0.25 mm i.d., 0.25 µm film thickness (Agilent). The carrier gas was helium at 1.0 mL/min constant flow. The injection volume was 1 µL or 2 µL. The initial oven temperature was 40 °C for 2 min. Then the temperature was ramped at 6 °C/min to 230 °C, which was held for 5 min. The end of the column was connected to a Deans switch (S+H Analytik; Mönchengladbach, Germany) used for heart-cutting. The Deans switch directed the eluate of the column via uncoated but deactivated fused silica capillaries (0.1 mm i.d.) time-programmed either simultaneously to the FID and the sniffing port used as monitor detectors or to a second GC column, which was a DB-1701 column, 30 m × 0.25 mm i.d., 0.25 µm film thickness (Agilent). This column was installed in a second Trace 1310 gas chromatograph. The capillary to the second column first passed through a heated (250 °C) hose connecting the two gas chromatographs and then through a liquid nitrogen-cooled trap used to refocus the heart-cut. The initial temperature of the second oven was 40 °C for 2 min. Then the temperature was ramped at 6 °C/min to 240 °C, which was held for 5 min. The end of the second column was connected to a Q Exactive GC orbitrap mass spectrometer (Thermo Fisher Scientific) operated in the high-resolution negative CI or positive CI mode with isobutane as reagent gas and a scan range of *m/z* 80–250. Data were analyzed with the Xcalibur software (Thermo Fisher Scientific).

Comprehensive Two-Dimensional GC×GC–MS Instrument

A 6890 Plus gas chromatograph (Agilent) was equipped with a GC PAL autosampler (CTC Analytics) and a CIS 4 injector (Gerstel; Mülheim an der Ruhr, Germany). The column in the first dimension was a DB-FFAP column, 30 m × 0.25 mm i.d., 0.25 µm film thickness (Agilent). The carrier gas was helium at 2.0 mL/min constant flow. The injection volume was 2 µL. The initial oven temperature was 40 °C for 2 min. Then the temperature was ramped at 6 °C/min to 230 °C, which was held for 5 min. The end of the first column was connected via a liquid nitrogen-cooled dual-stage quad-jet modulator (Leco; Mönchengladbach, Germany) to a DB-5 column, 3 m × 0.15 mm i.d., 0.30 µm film thickness (Agilent) inside the secondary oven, which was mounted inside the primary GC oven. The modulation time was 4 s. The initial temperature of the second oven was 70 °C for 2 min. Then the temperature was ramped at 6 °C/min to 250 °C, which was held for 5 min. The end of the second column was connected to a Pegasus III TOF mass spectrometer (Leco) operated in the electron ionization (EI) mode at 70 eV with a scan range of *m/z* 35–350 and a scan rate of 100 spectra/s. Data were analyzed with the GC Image software (GC Image; Lincoln, Nebraska, USA)

Table S1. Stable Isotopically Substituted Internal Standards, Quantifier Ions, and Calibration Lines Used in the Quantitation Assays

odorant	internal standard	quantifier ions (*m*/*z*)		calibration line equation[a]	R^2
		analyte	standard		
**3**	($^2H_3$)-**3**	131	134	y = 1.062x + 0.082	0.997
**6**	($^2H_2$)-**6**	81	83	y = 0.900x − 0.007	1.000
**10**	($^{13}C_8$)-**10**	111	119	y = 1.176x − 0.039	0.999
**11**	($^2H_4$)-**11**	127	131	y = 1.134x − 0.099	0.999
**12**	($^2H_2$)-**12**	125	127	y = 1.080x − 0.132	0.996
**16**	($^2H_3$)-**16**	105	108	y = 1.078x + 0.026	1.000
**17**	($^2H_2$)-**17**	157	159	y = 0.971x − 0.013	0.999
**22**	($^2H_3$)-**22**	171	174	y = 1.080x − 0.137	0.997
**23**	($^2H_2$)-**23**	89	91	y = 0.921x − 0.076	1.000
**27**	($^{13}C_2$)-**27**	139	141	y = 1.131x − 0.142	0.985
**28**	($^2H_3$)-**28**	171	174	y = 1.035x − 0.027	1.000
**31**	($^2H_2$)-**31**	137	139	y = 0.987x + 0.036	0.999
**32**	($^2H_{3–6}$)-**32**	191	194–197	y = 0.843x − 0.152	0.998
**33**	($^2H_2$)-**33**	137	139	y = 0.895x − 0.012	1.000
**35**	($^2H_2$)-**35**	183	185	y = 1.180x + 0.142	0.999
**37**	($^2H_2$)-**37**	97	99	y = 1.338x − 0.062	0.997
**38**	($^{13}C_6$)-**38**	137	143	y = 1.077x + 0.004	1.000
**40**	($^2H_{6–7}$)-**40**	109	115–116	y = 0.845x − 0.088	0.999
**42**	($^2H_2$)-MPP[b]	165	169	y = 0.854x − 0.096	0.995
**45**	($^2H_3$)-**45**	165	168	y = 0.953x + 0.025	1.000
**47**	($^2H_3$)-**47**	152	155	y = 1.057x + 0.079	1.000

[a]y = peak area counts standard / peak area counts analyte; x = concentration standard (μg/mL) / concentration analyte (μg/mL). [b]($^2H_2$)-2-methoxy-4-propylphenol.

Table S2. Concentrations of Important Odorants in Fresh Rhizomes of *H. cordata*

odorant	concentration (µg/kg)			
	experiment 1	experiment 2	experiment 3	mean ± SD (CV)[a]
**2a**	135000	143000	152000	143000 ± 8600 (6%)
**2b**	8590	9160	9690	9140 ± 550 (6%)
**3b**	0.159	0.140	0.147	0.149 ± 0.010 (6%)
**6**	15.6	14.6		15.1 ± 0.7 (5%)
**7**	104000	94300	136000	111000 ± 22000 (19%)
**8a**	30100	29600	33400	31000 ± 2000 (7%)
**8b**	10600	10400	11700	10900 ± 710 (7%)
**10**	42.8	34.8	42.2	39.9 ± 4.4 (11%)
**11**	4.08	5.47	4.54	4.70 ± 0.71 (15%)
**12**	1.29	1.11	1.08	1.16 ± 0.11 (10%)
**16**	6.72	7.64		7.18 ± 0.65 (9%)
**17**	5100	5630	4490	5070 ± 570 (11%)
**22**	1890	1770	2450	2040 ± 360 (18%)
**23**	1950	1700	1340	1660 ± 310 (18%)
**27**	≤0.0218[b]	≤0.0245[b]	≤0.0211[b]	
**28**	0.955	0.715	0.985	0.885 ± 0.148 (17%)
**31**	1570	2210	2350	2050 ± 410 (20%)
**32**	0.119	0.100	0.108	0.109 ± 0.009 (9%)
**33**	44.4	45.2	31.9	40.5 ± 7.4 (18%)
**35**	135	127	121	128 ± 7 (6%)
**36**	2380000	2410000	2660000	2480000 ± 160000 (6%)
**37**	0.0640	0.0736		0.0690 ± 0.0070 (10%)
**38**	1.37	1.58	1.37	1.44 ± 0.12 (8%)
**40**	6.02	6.70	6.55	6.42 ± 0.36 (6%)
**42**	56.4	57.8	63.1	59.1 ± 3.5 (6%)
**45**	15.5	16.2		15.9 ± 0.5 (3%)
**47**	148	153	145	149 ± 4 (3%)

[a]SD, standard deviation; CV, coefficient of variation. [b]No analyte peak was observed; values were derived from the integration of the background noise.

Table S3. Concentrations of Important Odorants in Fresh Leaves of *H. cordata*

odorant	concentration (μg/kg)			
	experiment 1	experiment 2	experiment 3	mean ± SD (CV)[a]
**2a**	5810	5400	7070	6090 ± 870 (14%)
**2b**	6820	6330	8300	7150 ± 1000 (14%)
**3b**	1.89	1.28	1.73	1.64 ± 0.32 (19%)
**6**	104	119	81.9	102 ± 19 (18%)
**7**	140000	137000	141000	140000 ± 1900 (1%)
**8a**	225	166	174	188 ± 32 (17%)
**8b**	4.59	3.38	3.56	3.84 ± 0.66 (17%)
**10**	24.7	22.6	24.9	24.1 ± 1.3 (5%)
**11**	4.64	5.45	6.06	5.38 ± 0.71 (13%)
**12**	1.13	0.976	1.04	1.05 ± 0.08 (7%)
**16**	0.106	0.143	0.186	0.165 ± 0.031 (19%)
**17**	12000	12300	16500	13600 ± 2500 (19%)
**22**	674	650	733	686 ± 43 (6%)
**23**	361	285	409	352 ± 63 (18%)
**27**	0.272	0.266	0.322	0.286 ± 0.031 (11%)
**28**	1.95	2.66	2.62	2.41 ± 0.40 (16%)
**31**	4910	5010		4960 ± 70 (1%)
**32**	0.874	0.727	0.879	0.826 ± 0.086 (10%)
**33**	3.96	4.08	4.86	4.23 ± 0.57 (13%)
**35**	85.1	108	83.2	91.9 ± 13.6 (15%)
**36**	1270000	1300000	1450000	1340000 ± 99000 (7%)
**37**	0.122	0.159		0.141 ± 0.026 (19%)
**38**	4.17	3.91	4.03	4.04 ± 0.13 (3%)
**40**	15.0	19.3	13.9	16.1 ± 2.8 (18%)
**42**	16.7	15.5	16.2	16.1 ± 0.6 (4%)
**45**	13.6	12.8		13.2 ± 0.6 (4%)
**47**	13.9	13.5	14.3	13.9 ± 0.4 (3%)

[a]SD, standard deviation; CV, coefficient of variation.
